# Ultrasound-stimulated microbubble radiation enhancement of tumors: Single-dose and fractionated treatment evaluation

**DOI:** 10.1371/journal.pone.0239456

**Published:** 2020-09-25

**Authors:** Evan McNabb, Azza Al-Mahrouki, Niki Law, Scott McKay, Christine Tarapacki, Farah Hussein, Gregory J. Czarnota

**Affiliations:** 1 Physical Sciences, Sunnybrook Research Institute, Toronto, Ontario, Canada; 2 Leslie Dan Faculty of Pharmacy, University of Toronto, Toronto, Ontario, Canada; 3 Department of Radiation Oncology, Sunnybrook Health Sciences Centre, Toronto, Ontario, Canada; 4 Department of Chemistry, University of Manitoba, Winnipeg, Manitoba, Canada; 5 Department of Physics, Western University, London, Ontario, Canada; 6 Department of Radiation Oncology, University of Toronto, Toronto, Ontario, Canada; 7 Department of Medical Biophysics, University of Toronto, Toronto, Ontario, Canada; Columbia University, UNITED STATES

## Abstract

The use of ultrasound-stimulated microbubble therapy has successfully been used to target tumor vasculature and enhance the effects of radiation therapy in tumor xenografts in mice. Here, we further investigate this treatment using larger, more clinically relevant tumor models. New Zealand white rabbits bearing prostate tumor (PC3) xenografts received a single treatment of either ultrasound-stimulated microbubbles (USMB), ionizing radiation (XRT; 8Gy), or a combination of both treatments (USMB+XRT). Treatment outcome was evaluated 24 hours after treatment using histopathology, immunolabeling, 3D Doppler ultrasound and photoacoustic imaging. A second cohort of rabbits received multiple treatments over a period of three weeks, where USMB treatments were delivered twice weekly with daily XRT treatments to deliver a fractionated 2Gy dose five days per week. A significant decrease in vascular function, observed through immunolabeling of vascular endothelial cells, was observed in tumors receiving the combined treatment (USMB+XRT) compared to control and single treatment groups. This was associated with an increase in cell death as observed through in situ end labeling (ISEL), a decrease in vascular index measured by Power Doppler imaging, and a decrease in oxygen saturation. In rabbits undergoing the long-term fractionated combined treatment, a significant growth delay was observed after 1 week and a significant reduction in tumor size was observed after 3 weeks with combined therapy. Results demonstrated an enhancement of radiation effect and superior anti-tumor effect of the combination of USMB+XRT compared to the single treatments alone. Tumor growth was maximally inhibited with fractionated radiotherapy combined with the ultrasound-stimulated microbubble-based therapy.

## Introduction

Approximately half of cancers are treated with radiation therapy in their management and extensively in the treatment of breast, head and neck, lung and prostate cancers [1–3]. In addition to canonical DNA damage, radiation affects tumors and their microenvironment through reoxygenation, vascular density and permeability changes, blood flow alterations, and changes interstitial fluid pressure [4–11]. Radiation therapy specifically can lead to vascular endothelial cell death and changes to the tumor vascular network, which influence therapeutic outcomes in a dose-dependent manner [12–14]. In order to improve its efficacy, radiation therapy is often administered in combination with other treatments to increase radiosensitivity, for example in conjunction with chemotherapy, nanoparticles, and molecular-targeted therapies [15–20]. These often introduce systemic effects, often leading to difficulty in targeting the local tumor microenvironment.

Recent investigators have developed a novel and effective combined treatment approach where intravenous microbubbles are stimulated using ultrasound waves to perturb the tumor vascular endothelial cells resulting in enhanced effects of radiation [21, 22]. Microbubbles are composed of a gas core (often perfluorocarbon), and a lipid or polymer shell on the order of 1–3 μm in diameter, and find clinical use as an ultrasound contrast agent for visualizing perfusion [23, 24]. Upon exposure to ultrasound the bubbles may undergo oscillation and cavitation, transferring mechanical stress to nearby endothelial cell membranes [25]. The cell membrane effect sensitizes the endothelial cells to radiation treatment through a membrane-activated, ceramide-mediated cell death pathway that would otherwise only be activated at very high single doses of radiation, i.e., > 10 Gy [26–28].

Investigations have demonstrated that USMB treatments, when combined with low doses of radiation, result in significant tumor size reduction [21]. Work has investigated the level of disruption of endothelial cells *in vitro* and identified potential gene markers and signalling pathways implicated in the response to this therapy [29, 30]. Specifically, results have demonstrated that endothelial cell death in response to this treatment is predominantly dependent on ceramide production through acid sphingomyelinase (ASMase) signalling [31, 32]. Furthermore, acute vascular shutdown and significant tumor cell death is observed [33, 34], indicating that tumor cell death is secondary to endothelial cell death. Several *in vivo* studies have also indicated the effectiveness of this therapy in treating xenograft tumors in murine models, including breast, prostate liver, and bladder [21, 22, 35–40].

The work here is the first to investigate the treatment of a larger animal cancer model using ultrasound-stimulated microbubbles radiation enhancements. In this study, we demonstrate that ultrasound-stimulated microbubble treatments significantly enhance tumor cell death when combined with radiation therapy. Specifically, rabbits bearing human PC-3 tumor xenografts in the hind leg received combined treatments of USMB and radiation. Both in the case of single high-dose radiation treatments and multiple fractionated radiation treatments, tumor cell death was enhanced significantly through the ultrasound-based approach. Results further indicated that the enhancement of tumor cell death was correlated with vascular disruption.

## Materials and methods

### Animal handling and cell culture

All animal experiments were approved by the Sunnybrook Research Institute Animal Care Committee (SRI ACC) and were conducted in compliance with the approved animal utilization protocol guidelines (protocol #539) and internationally recognized guidelines from the Canadian Council on Animal Care (CCAC). All surgeries and interventions were performed under ketamine and xylazine anaesthesia, and all efforts were made to minimize suffering. Our study utilized the following humane endpoints: weight loss of more than 20% or lack of feeding, dragging tumor-bearing legs and other lack of ambulation, tumors exceeding 2 cm in diameter, and self-mutilation, bleeding or ulcerations exceeding 20% of the tumor area. Animals were evaluated in-house daily by trained veterinary staff and supportive care (analgesia and polytopic antibiotics) was given when necessary for minor wounds. Animals were housed individually, provided fresh food and water daily, and all cages are supplied with enrichment items. Daily checks to animal weight, sickness, wounds, excrement, etc. were scored and recorded. All animals were euthanized under anaesthesia with intravenous sodium pentobarbitol (Euthanyl) immediately at endpoints.

Fifty-five New Zealand white rabbits (1.80–3.34 kg, Charles River Laboratories, Montreal, QC, Canada) received three treatments of enrofloxacin (Baytril) at 5 mg/kg upon arrival, and were housed locally in HEPA filtered, positive pressure clean rooms until they reached the appropriate weight range listed (approximately 7 to 10 days). PC3 tumor cell preparations were injected into the right hind leg of the rabbits using a 27-gauge needle. Tumors were allowed to grow until 1.5–2.0 cm in diameter over a period of three to four weeks. The rabbits were immunosuppressed by daily intramuscular injections of cyclosporine at 50 mg/mL (Sandimmune, Novartis, Dorval, QC, Canada). One animal reached humane endpoint during the tumor growth period (tumor ulceration) and was euthanized, while a second expired under anaesthesia during therapy. A total of fifty-three animals were used for the remainder of the study.

Prostate cancer (PC3) cells were purchased directly from a vendor (ATCC CRL1435, Manassas, VA, USA) and were cultured in RPMI 1640 media (Wisent Bio-Centre, St-Bruno, QC, Canada) and supplemented with 10% FBS and 1% penicillin/streptomycin (Thermo Fisher Scientific, Waltham, MA, USA). Cell cultures were incubated at 37C and 5% CO2. Cell suspensions were prepared in phosphate buffered saline (PBS) for tumor induction from cell culture passages 3 to 6. Each preparation contained 7×10^6^ cells in 700 μL.

### Experimental setup

The first cohort for experimentation consisted of 31 rabbits, which were placed into the following treatment groups: control (no treatment; n = 7), ultrasound-stimulated microbubble therapy (USMB; n = 5), radiation therapy (XRT; n = 12), and combined treatment of ultrasound-stimulated microbubble therapy followed by radiation therapy (USMB+XRT; n = 7). Minimum experimental group sizes were calculated (n = 4) from the minimum effect size in murine models at the selected radiation and USMB therapy parameters to ensure the statistical power (1 - β) > 0.8 [21]. Rabbits were sedated using a combination of 50 mg/kg of ketamine and 5 mg/kg of xylazine injected intramuscularly. The rabbits were placed on an acrylic platform mounted on top of a 37° C water bath. The tumor-bearing leg was moved off the platform and into the water. The ultrasound transducer was then vertically aligned to match the position of the tumor on the leg. Definity microbubbles (Lantheus Medical Imaging, Billerica, MA, USA) were brought to room temperature and activated using a Vialmix device (Lantheus Medical Imaging) for 45 seconds. A 1 mL solution of bubbles was diluted with 2 mL of saline, resulting in a 1% (v/v) concentration relative to the mean rabbit blood volume. The entire 3 mL volume was injected intravenously into the ear and flushed with an additional 3 mL saline (supplemented with 0.2% heparin) immediately prior to sonication.

Ultrasound stimulation was delivered using an in-house built system which included a waveform generator (AWG520, Tektronix, Beaverton, OR, USA), a pulser/receiver (RPR4000, Ritec, Rochster, NY, USA), and a transducer with a 500 kHz central frequency (Valpey Fisher Inc., MA, USA). The focal point of the transducer’s -6 dB beam width was 31 mm and was located 8.5 cm from the scan head. Ultrasound was emitted at a peak negative pressure of 565 kPa using a pulse sequence consisting of a 16-cycle tone burst with 3 kHz pulse repetition frequency for 5 minutes. The radiation treatments were administered as a single 8 Gy dose using a 160 kVp cabinet irradiator (CP160 X-Ray Irradiation System, Faxitron Bioptics, Tuscon, AZ, USA). During radiation treatments, the rabbits were shielded with a 3 mm thick lead sheet, while the tumor was exposed through a circular cut-out. For rabbits receiving the combined treatments, the microbubble treatments were administered first, followed immediately by radiation.

A multi-fraction treatment study was performed in a second cohort of 22 rabbits using the same treatments conditions as above. Humane endpoints were reached in 3/22 animals (lack of ambulation, weight loss or lack of feeding, and ulcerations, respectively). Two did not recover from anaesthesia. The groups were control (no treatment; n = 5), ultrasound-stimulated microbubble therapy (USMB; n = 3), radiation therapy (XRT; n = 7), and combined treatment of ultrasound-stimulated microbubble therapy followed by radiation therapy (USMB+XRT; n = 7). In contrast, the USMB treatments were administered twice weekly with the same microbubble solution and ultrasound parameters as above. Radiation treatments were delivered using a non-curative fractionated schedule of 2 Gy/day administered 5 days per week for a three-week period for a total dose of 30 Gy in 15 fractions (BED_10_ = 36 Gy). Changes in tumour sizes were measured using a Vernier caliper prior to treatment as an individual baseline, and subsequently at the end of each week of treatment.

### Power Doppler and photoacoustic imaging

Power Doppler images were acquired using a VEVO 770 system (VisualSonics, Toronto, ON, Canada) with a 25 MHz transducer (RMV-710B: 20 MHz centre frequency, 2.5 mm/s wall filter, 2.0 mm/s scan speed, 0.2 mm step size). Power Doppler images were analyzed using in-house software (MATLAB, Mathworks, Natick, MA) to calculate the vascular index (VI) for each tumor (described in [40]). The relative change in VI (VI_post_—VI_pre_)/ VI_pre_ was determined in order to normalize the data for each rabbit and quantitatively assess changes in perfusion. Photoacoustic (PA) images were acquired on a VEVO 2100 system using a 15 MHz transducer (LZ-201: 15 MHz centre frequency, 43 dB gain, 0.2 mm step size). The photoacoustic oxygen saturation information was acquired using both 750 nm and 850 nm wavelengths and the “oxy-hemo” 3D imaging mode. PA images were analyzed using the VisualSonics 2100 software to determine the level of oxygen saturation. The single-fraction treatment cohort was imaged prior to treatment and 24-hours post treatment. The multi-fraction treatment cohort was imaged before treatment on the first day of the study and weekly thereafter.

### Histopathology

Each tumor in the first cohort was excised 24-hours after treatment. This sacrifice time point has previously demonstrated DNA fragmentation, apoptosis and ISEL-positive cell kill for single-exposure experiments [21, 30]. The tumor tissue was sliced in half axially to match the imaging position. One half was fixed in a 10% neutral buffered formalin solution (Fischer, Toronto, ON, Canada) and the second half was embedded in an optimal cutting temperature compound (O.C.T., VWR, Toronto, ON, Canada), then snap frozen in liquid nitrogen and stored at -80° C. The formalin-fixed portion of the tumor was dehydrated, embedded in paraffin wax, and sectioned into 5 mm thick slices. Tumor sections were stained with hematoxylin and eosin (H&E) to visualize the histopathology. DNA fragmentation was identified using in situ end labeling (ISEL). Changes in vasculature were detected via immunolabeling of the vascular marker factor VIII (described in ref. [41]). Staining for fibrosis was conducted using a Richard-Allan Scientific Chromaview kit (Thermo Fisher Scientific), which stains red for cytoplasm, keratin and muscles; blue for mucin and collagen; and, black for nuclei. Quantification of ISEL, factor VIII, and trichrome staining were conducted using ImageJ (National Institutes of Health, Bethesda, MD, USA). For whole-slide analysis (1X or 0.8X), image masks were created from the confirmed tumor areas on the H&E stained sections and then applied to either the ISEL or trichrome stains to exclude skin, muscle, and necrosis. The total surface area with positive staining was measured relative to the total surface area examined. For each of the factor VIII stained sections, five random regions of interests within the confirmed tumor area, approximately 0.1 mm^2^, were selected and viewed at 20X magnification and digitized. In the second cohort of animals, histopathology was obtained once an endpoint was reached.

### Statistical analysis

Quantifiable parameters from the single fraction treatment cohort (VI, %SO_2_, cell death and vascularity staining) were compared for each of the four groups using a one-way analysis of variance (ANOVA). Group-wise comparisons were performed with Tukey multiple comparison corrections. The multi-fraction treatment cohort used a repeated measures two-way ANOVA to test the tumor size by treatment and time. Each rabbit had four repeated measurements at baseline, and weeks 1 through 3. Survival analysis was performed using a log-rank (Mantel-Cox) test and calculations were performed in Graphpad Prism 6.0 (Graphpad Software, San Diego, CA, USA). All six survival pair-wise group comparisons were performed controlling the false discovery rate (*q* = 0.05), and multiplicity adjusted p values reported. Quantitative values reported values were the mean, standard error, and 95% confidence intervals unless otherwise stated. All statistical tests were two-sided, and a p-value less than 0.05 was considered statistically significant.

## Results

### Single-dose USMB and XRT treatment experiments

#### Histopathology of cell death and vascular density

**Fig 1:**
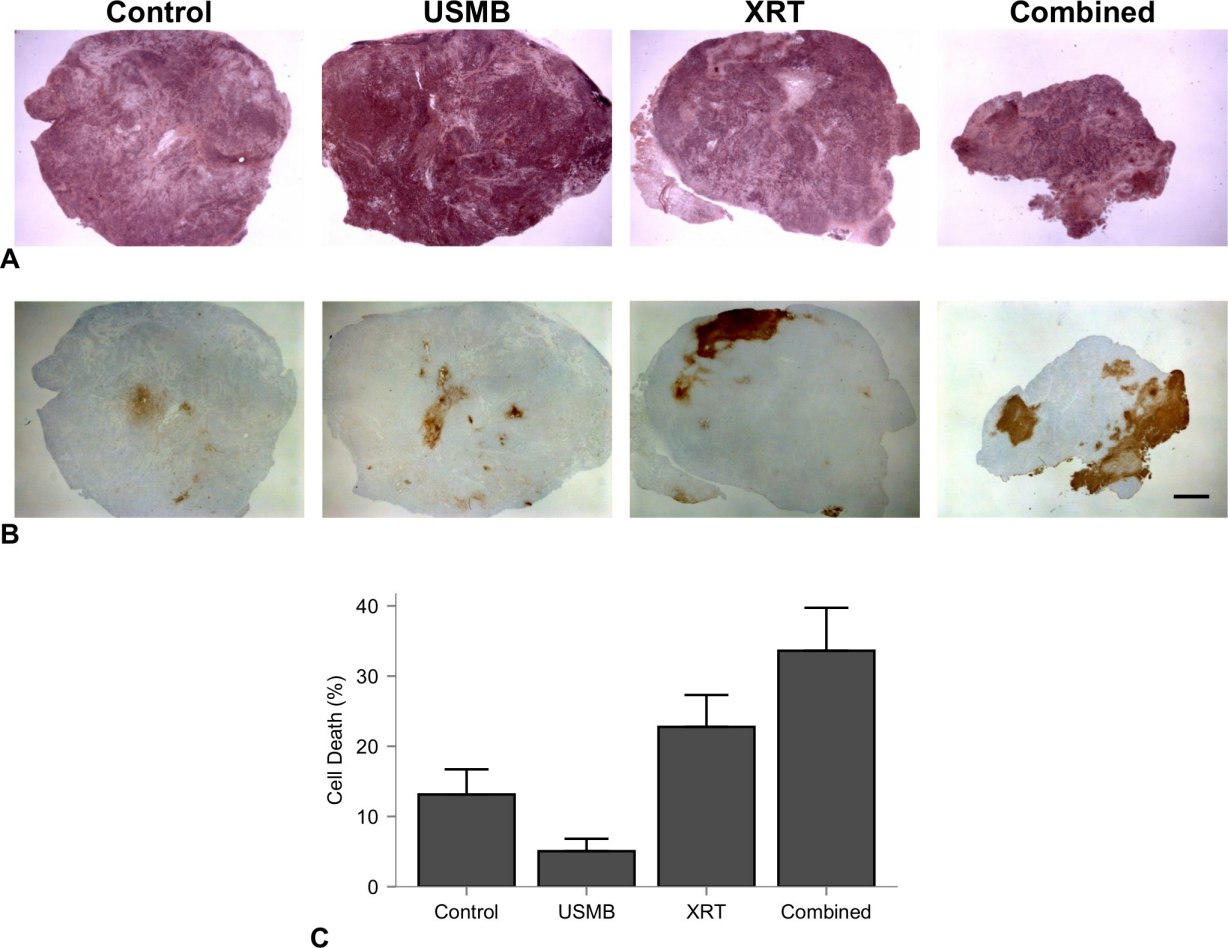
Representative hematoxylin and eosin and corresponding in situ end labelling (ISEL)-stained sections. (A) Representative hematoxylin and eosin and corresponding in situ end labelling (ISEL)-stained. (B) sections of PC3 prostate tumors treated with ultrasound-stimulated microbubbles and/or radiation. Columns represent untreated tumors (control), 1% (v/v) microbubble and focused ultrasound exposure (USMB), 8 Gy radiation exposure (XRT), and combined treatments. Microbubble alone or radiation alone exposures resulted in minor localized areas of cell death, and the combination resulted in larger detectable areas of cell death, appearing as clear zones in the hematoxylin and eosin-stained slides and brown areas in the ISEL-stained slides. Scale bar is 2 mm. (C) Quantified percent cell death for each treatment group. Error bars represent SEM.

Results indicated that the combination of USMB treatment and radiation resulted in enhanced tumor cell kill and vascular disruption. Representative hematoxylin and eosin and corresponding in situ end labelling (ISEL)-stained slides are shown in Fig 1A and 1B, after 24 hours following treatments of a single dose of 8 Gy radiation, USMB treatment, or combinations of treatment. The cell death measurements were 13 ± 9%, 5 ± 4%, 23 ± 16%, and 34 ± 16% for the control, USMB, XRT, and combined (USMB+XRT) treatments, respectively (Fig 1C). Analysis (ANOVA) indicated a significant difference between the groups in terms of cell death (*p* = 0.0057). The combined treatment had significant mean differences from ISEL+ stained cell death of 20 ± 7% (CI: 0.78 to 40%, *p* = 0.039) relative to control, and 29 ± 8% (CI: 6.98 to 50%, *p* = 0.006) relative to USMB treatments alone.

**Fig 2:**
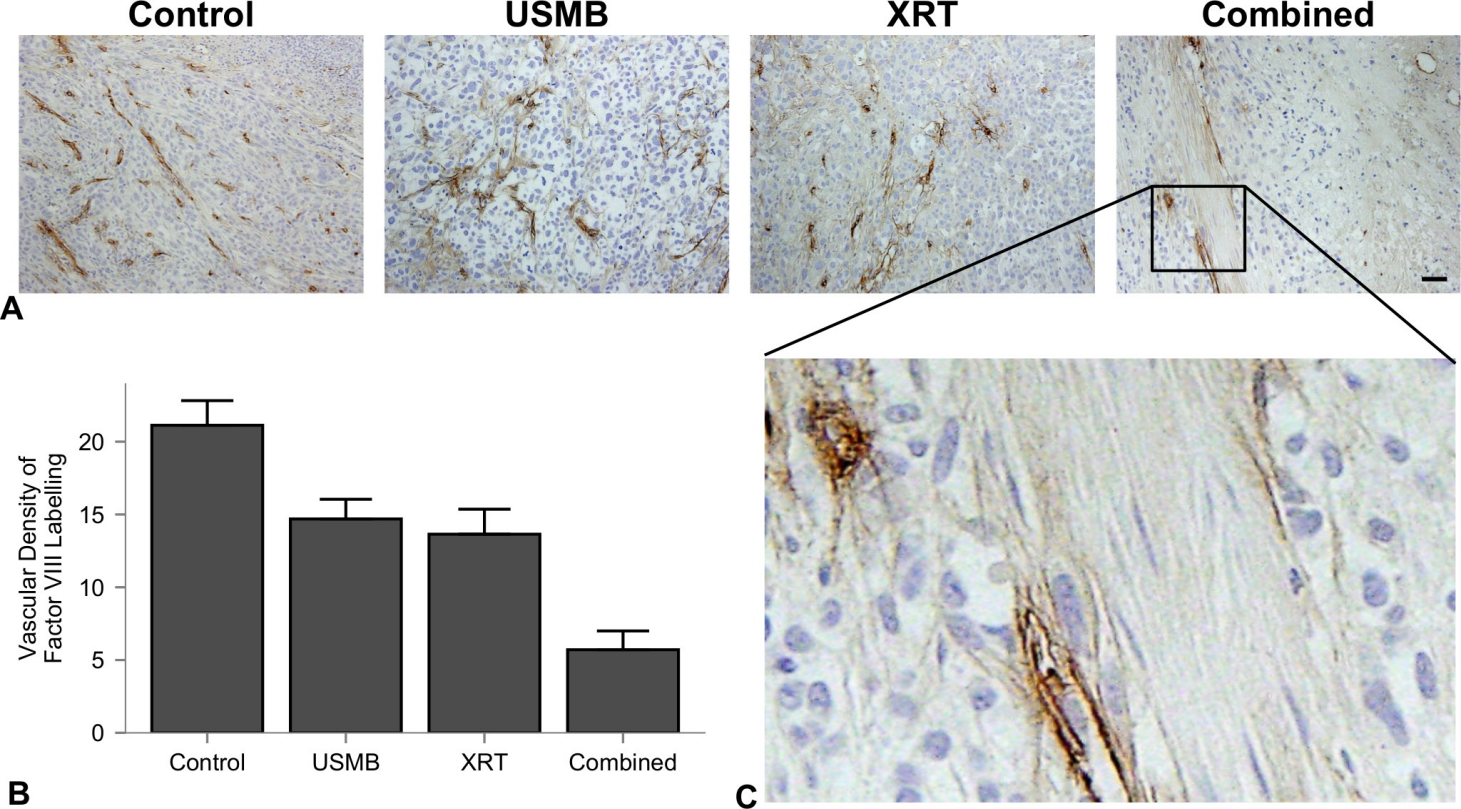
Factor VIII-stained sections of untreated tumors, 1% (v/v) USMB exposure, 8 Gy radiation exposure and combined treatments. (A) Representative Factor VIII-stained sections. Columns represent untreated tumors, 1% (v/v) microbubble and focused ultrasound exposure (USMB), 8 Gy radiation exposure (XRT), and combined treatments. Tumor sections (scale bar: 50 um) from all treatments exhibit a reduction in blood vessel density. (B) Quantified vascular density measurements. Error bars represent SEM. All groups have statistically significant mean differences except for USMB vs XRT only treatments. (C) Enlarged section of a combined treatment slide at 10X relative magnification displaying positive stained blood vessels for counting the vascular density.

Representative Factor VIII-stained sections displaying vascular density in response to treatment are presented (Fig 2A). Quantification of the vascular density is presented (Fig 2B), where analysis (ANOVA) indicated a highly significant difference between the groups (*p* < .0001). Overall, tumors treated with USMB and radiation demonstrated significant decreases in vascular density compared to controls. The value for the control group was 23 ± 5 vessels/0.1 mm^2^, which dropped to 15 ± 3 (*p* = 0.027) and 14 ± 5 (*p* = 0.002) for the USMB-alone and radiation-alone treatments, respectively. No significant differences were observed between these two treatments. In contrast, the combined group had a significant decrease in vascular density at 6 ± 3 vessels/0.1 mm^2^. The combined treatments significantly differed from all other groups, with mean differences of -17 ± 2 (CI: -23 to -10, *p* < 0.001) relative to control, -9 ± 3 (CI:-16 to -2, *p* = 0.011) relative to USMB alone, and -8 ± 2 (CI: -14 to -2, *p* = 0.007) relative to radiation treatment alone.

#### Power Doppler and photoacoustic imaging

**Fig 3:**
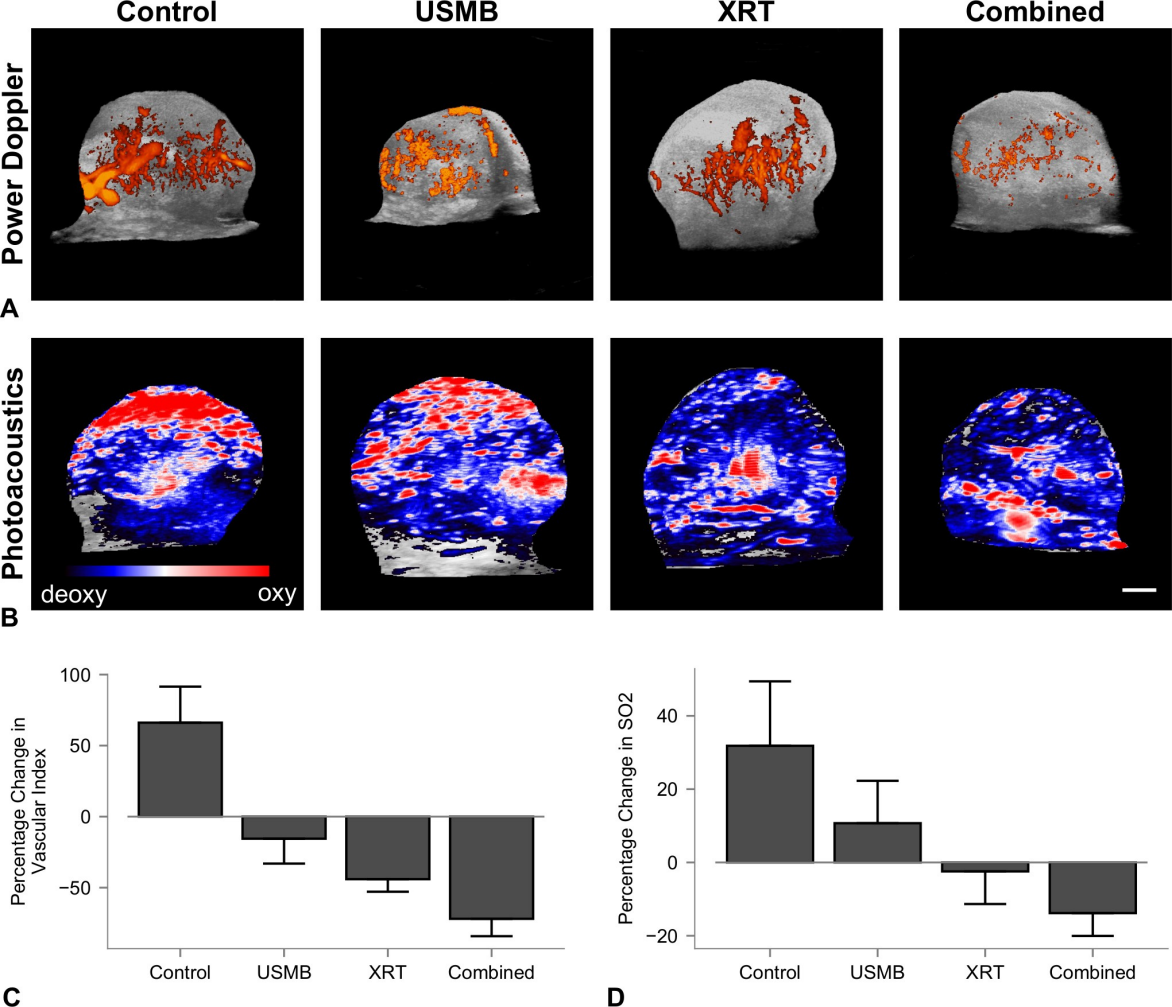
Volumetric 3D power Doppler depicting blood flow and photoacoustic imaging depicting changes in oxygen saturation. (A) Maximum intensity projections of volumetric 3D power Doppler depicting blood flow 24 hours after treatment, (B) and photoacoustic imaging depicting the changes in oxygen saturation. Coloured overlays represent 0–40 dB for power Doppler imaging, and blue-red colour bar represents relative ratio of oxy- and deoxyhemeglobin levels. Columns represent untreated tumors, 1% (v/v) microbubble and focused ultrasound exposure (USMB), 8 Gy radiation exposure (XRT), and combined treatments. The scale bar represents 4 mm. (C and D) Quantified percentage change in VI and oxygen saturation. Error bars represent SEM.

Representative maximum intensity projections of volumetric tumor images obtained with 3D power Doppler are presented (Fig 3A). These images qualitatively depict the intensity of blood flow 24 hours after treatment. The vascular index (VI) was used to quantify the relative changes within tumors, normalized to each rabbit's pre-treatment power Doppler data (Fig 3C). Analysis (ANOVA) revealed a significant difference between the treatment groups (*p* = 0.0003). The changes detected were 66 ± 25%, -15 ± 18%, -44 ± 9%, and -71 ± 12% for control, USMB-alone, XRT-alone, and combined treatment groups, respectively. Each treatment had a significant difference in the percentage change in VI relative to the control group of -82 ± 24% (CI: -153 to -10%, *p* = 0.023) for USMB-alone, -110 ± 24% (CI: -181 to -39%, *p* = 0.003) for XRT-alone, and -138 ± 23% (CI: -205 to -70%, *p* = 0.002) for combined treatments. However, there were no significant differences between individual treatment group types.

Photoacoustic (PA) imaging was used to assess the changes %SO_2_. The percentage changes were 32 ± 35%, 11 ± 23%, -2 ± 18%, and -14 ± 14% for control, USMB-alone, XRT-alone, and combined USMB+XRT treatment groups respectively (Fig 3B). Relevant decreases in oxygen saturation were found between each of the treatment groups and control, with the largest difference of -46 ± 16% (CI: -91 to 0.2%, P = 0.051) from the combined treatment. However, no significant differences between the treatments and control, or among the treatment groups, were found.

### Multi-fraction treatment experiments

**Fig 4:**
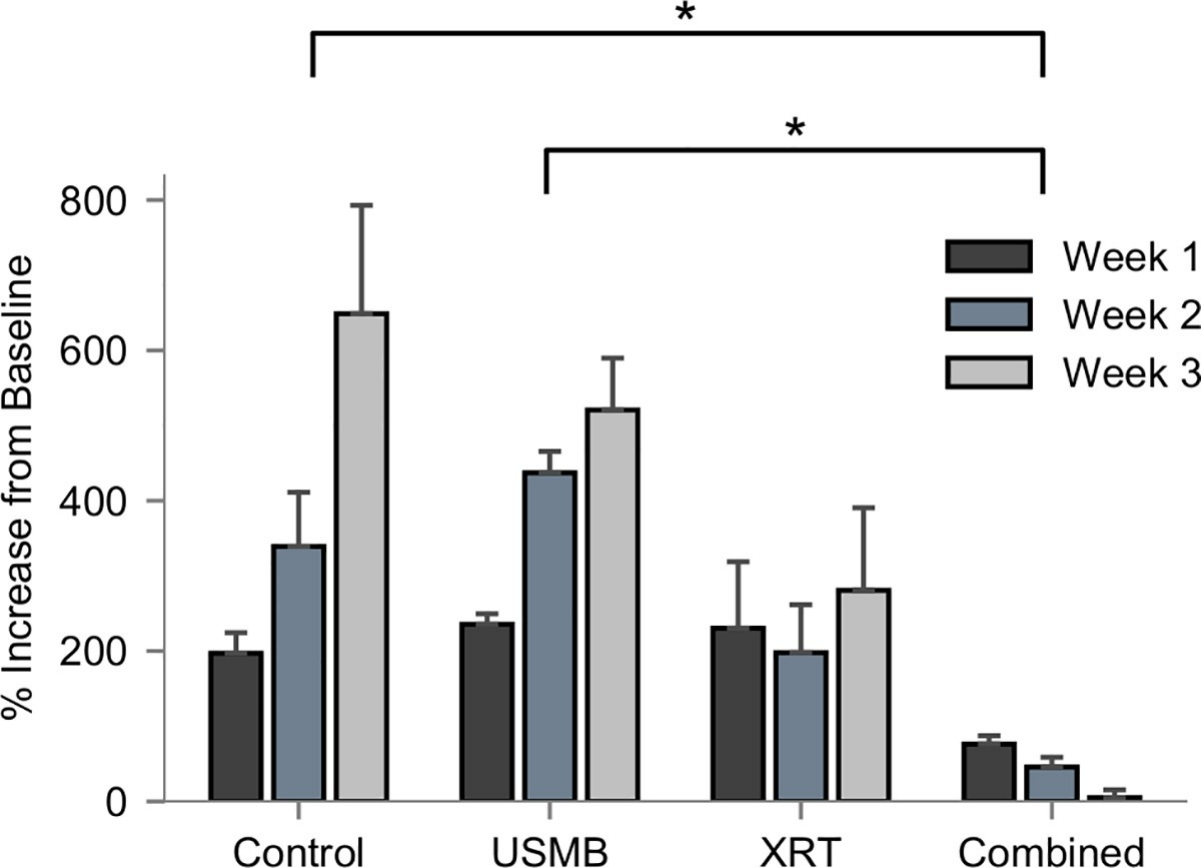
Weekly measurements of tumor volume (%mm3) relative to pre-treatment baseline values expressed as percentage change. Groups represent untreated tumors, 1% (v/v) microbubble and focused ultrasound exposure (USMB), multi-fraction radiation (XRT), and combined treatments. The USMB treatments were administered twice weekly, and radiation in the XRT and combined treatments were administered in five fractions/week at 2 Gy each over a 3-week period (BED10 = 30 Gy). Analysis (two-way ANOVA) revealed significant differences by treatment (p = 0.0008) and an interaction between measurement time and treatment (p < .0001).

In order to further investigate the ability of ultrasound-stimulated microbubble therapy to enhance the effects of radiation, we tested a fractionated low-dose regimen with the same treatment types overall. The radiation in the XRT-alone and combined therapies were administered at 2 Gy, five times per week over a 3-week period, resulting in a total biological effective dose (BED_10_) of 36 Gy. Analysis (ANOVA) indicated a significant effect in time as well as interaction between time and treatment group type (both *p* < .0001). Tumors in the control group continued to grow rapidly with a significant increase in tumor size between weeks 1 and 3 of +435% (CI: 288 to 582%, *p* < .0001) relative to their baseline values. Similarly, USMB only treated tumors also continued to grow between weeks 1 and 3 of +285% (CI: 116 to 454%, *p* = 0.0008). Tumors treated with XRT exhibited slower, non-significant growth over the three-week period, and those treated with the combined therapy had negative growth for each weekly comparison. Significant reductions in tumor sizes were observed per group (*p* = 0.0081). The combined therapy resulted in a group-wise mean differences of -372 (CI: -660 to -84%, *p* = 0.0105) relative to control, and -355% (CI: -669 to -42%, *p* = 0.0245) relative to USMB-alone (Fig 4). Finally, the mean differences for all weekly tumor sizes are shown further in Table 1.

**Table 1:** Mean differences (% change mm^3^) in tumor size relative to each rabbit’s baseline.

	Control			USMB		
Week	Mean Diff.	95% CI	p-value	Mean Diff.	95% CI	p-value
2 vs 1	168%	20.9% to 314%	0.0228	201%	32.2% to 370%	0.0173
			*			*
3 vs 1	435%	288% to 582%	< 0.0001	285%	116% to 454.3%	0.0008
			****			***
3 vs 2	268%	121% to 414%	0.0003	83.6%	-85.6% to 253%	0.4477
			***			
	**XRT**			**Combined (USMB+XRT)**		
**Week**	Mean Diff.	95% CI	p-value	Mean Diff.	95% CI	p-value
2 vs 1	-40.1%	-171% to 91.0%	0.7303	-47.7%	-179% to 83.4%	0.6428
3 vs 1	42.7%	-88.5% to 174%	0.7002	-79.7%	-211% to 51.4%	0.3028
3 vs 2	82.8%	-48.2% to 214%	0.2761	-33.0%	-163% to 99.1%	0.8179

p < 0.05 used for each test. Tukey correction for multiple comparisons was used.

df = 3 (group), 2 (time), 6 (interaction), 13 (subject), 26 (residual).

**Fig 5:**
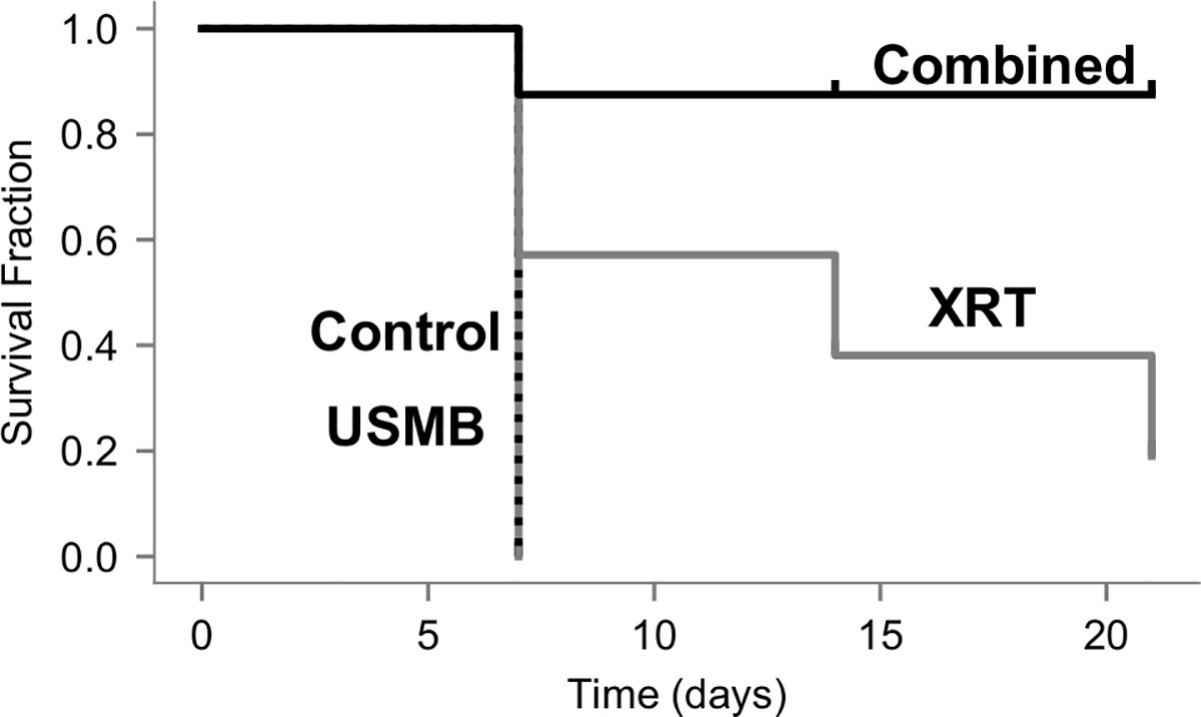
Kaplan-Meier survival curves are presented for survival assessment. Groups represent untreated tumors (control), 1% (v/v) microbubble and focused ultrasound exposure (USMB), multi-fraction radiation (XRT), and combined treatments. The USMB treatments were administered twice weekly, and radiation in the XRT and combined treatments were administered in five fractions/week at 2 Gy each over a 3-week period (BED10 = 30 Gy). Endpoints were rabbit death, tumors doubling in size or modified human endpoints. A log-rank test revealed significant differences among the curves (*p* = 0.002). Pair-wise comparisons were made controlling the false discovery rate to 5%. The mean survival after one week diminished to zero for untreated and USMB treatments, respectively. The XRT group diminished to 19% by the end of the third week. The combined group dropped to 88% after the first week where it remained for all subsequent weeks and was statistically significant compared to all other treatment groups.

Analysis (log-rank Mantel-Cox) of survival curves to modified human endpoint, or tumor doubling in size revealed significant differences from the treatments (*p* = 0.002), shown below (Fig 5). The surviving fractions for the XRT-alone and combined 2 Gy and USMB treatments were 14% and 87% after 21 days, respectively. The control and USMB-alone treatment groups did not have surviving fractions as modified human endpoints were exceeded after 7 days. Pair-wise comparisons (log-rank Mantel-Cox) controlling false discovery rate at 5% demonstrate that combined treatment significantly differed from the control (*p* = 0.019), USMB only (*p* = 0.028), and XRT only (*p* = 0.021) treatments, respectively. The XRT only treatment did not significantly differ from the control or USMB treatments (*p* = 0.16).

**Fig 6:**
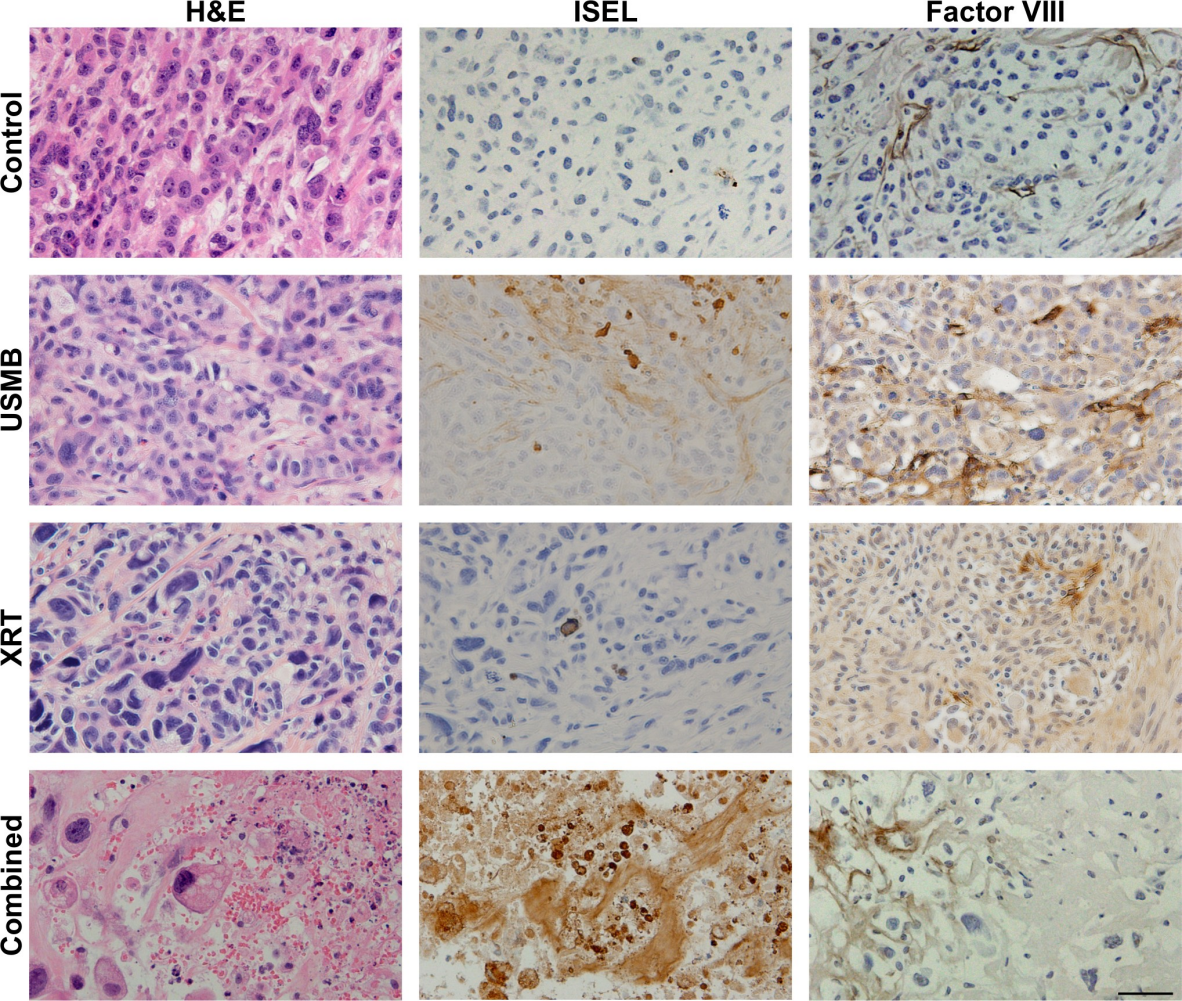
Representative H&E, and corresponding ISEL- and Factor VIII-stained slices at the end of week 3. Rows represent untreated tumors, 1% (v/v) microbubble and focused ultrasound exposure (USMB), multi-fraction radiation (XRT), and combined treatments. The USMB treatments were administered twice weekly, and radiation in the XRT and combined treatments were administered in five fractions/week at 2 Gy each over a 3-week period (BED10 = 36 Gy). Relative to control, the USMB and XRT treatments do not show appreciable cellular death, however the combination of the treatments caused the largest detectable ISEL stained areas (diffuse brown staining in ISEL column). Vascular disruption was most prominent with the combined treatment with fewer intact blood vessels. Scale bar represents 50 um.

**Fig 7:**
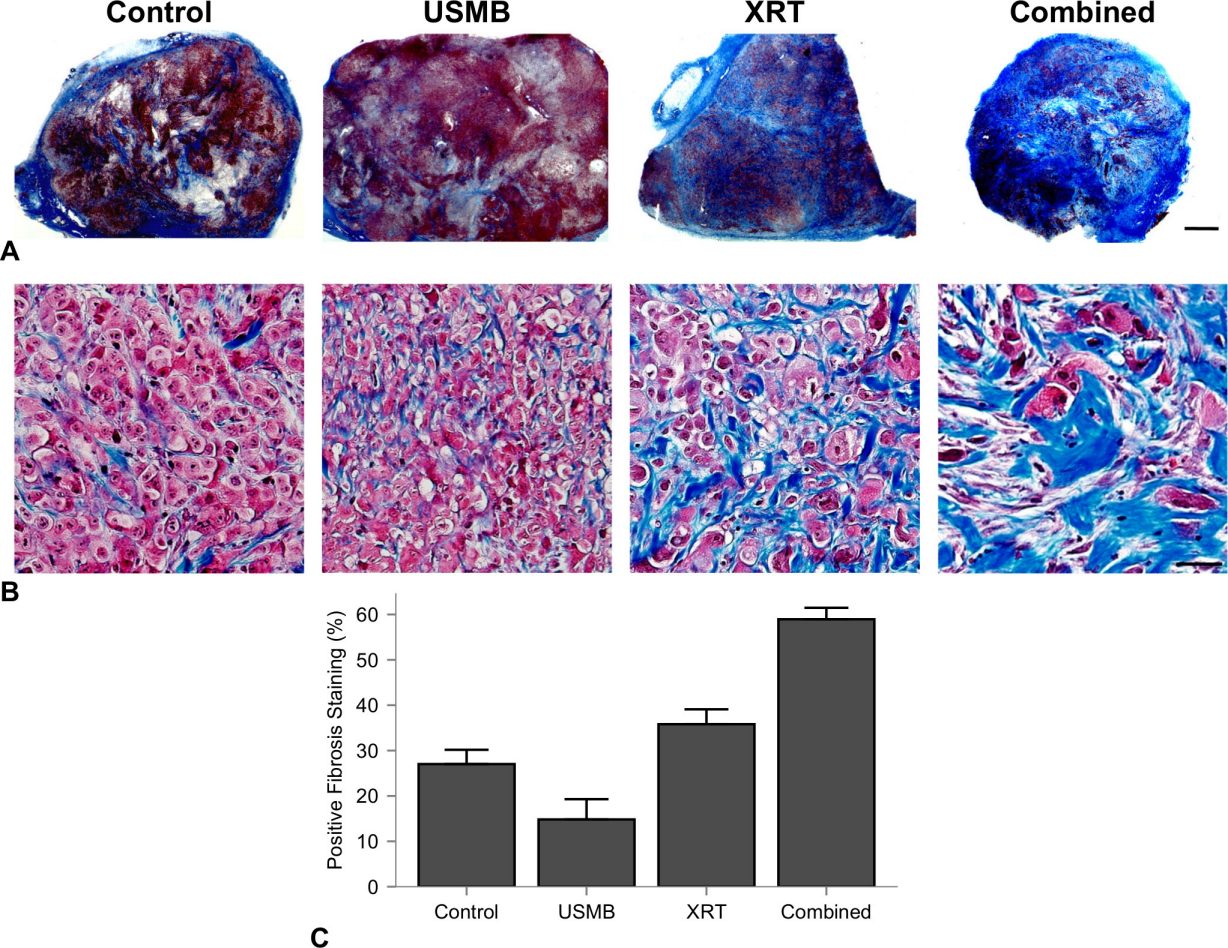
Representative trichrome-stained whole mount sections of each treatment group at the end of week 3. (A) Representative trichrome-stained whole mount sections of each treatment group at the end of week 3. Columns represent untreated tumors, 1% (v/v) microbubble and focused ultrasound exposure (USMB), multi-fraction radiation (XRT), and combined treatments. The USMB treatments were administered twice weekly, and radiation in the XRT and combined treatments were administered in five fractions/week at 2 Gy each over a 3-week period (BED10 = 30 Gy). Blue labelled regions increased by the third week in tumors that received the combined treatment. Scale bar is equal to 2 mm. (B) Higher magnification indicate the increase in fibrotic tissue replacing previous cell death. Scale bar represents 50 um. (C) Quantified percent cell death for each treatment group. Error bars represent SEM. These data indicate an enhancement in trichrome stained regions.

Representative labelled slides of each treatment group are presented (Fig 6). H&E, ISEL and factor VIII staining from the control group demonstrated viable tumor cells and intact vasculature. The USMB-only treatment tumor specimens indicated little to no tumor cell death, and intact vasculature. The XRT only treatments indicated mostly viable cells throughout the tumor, however the vascular network was sparse, specifically in the factor VIII staining. The combined treatment tissues were visibly affected, as ISEL indicated tumor cell apoptosis and factor VIII staining indicated disruption of the vascular network. Whole mount tumor sections were stained for fibrosis using a trichrome staining kit are displayed (Fig 7). The presence of collagen fibres was minimal in the control and USMB only treatment groups, and the surrounding tumor cells appeared viable. Tumors that received fractionated XRT and combined radiation present with increased fibrosis (blue coloured sections) and areas of cell death, suggesting that viable tumor areas are being replaced with fibrotic tissue (Fig 7B). Treatment with USMB, XRT, and USMB+XRT demonstrated changes in fibrotic staining of -12 ± 4.9% (*p* > 0.05), 8.8 ± 5.0% (*p* > 0.05), and 32 ± 5.0% (CI: 18 to 45%, *p* < .0001), respectively, in comparison to untreated control tumors (Fig 7C). Analysis (ANOVA) revealed a significant difference between the treatment groups (*p* < .0001). Combined USMB+XRT treatments had significant increases in staining of 44.12 ± 4.8% (CI: 30 to 58%, *p* < .0001) to the USMB-alone group, and 23 ± 4.5% (CI: 10 to 37%, *p* = 0.0015) to the XRT-alone group. No significant differences between USMB-alone or XRT-alone in comparison to untreated control tumors. Regression analysis which included a two-way interaction between USMB and XRT revealed a significant positive coefficient (*p* = 0.0002).

## Discussion

The work here demonstrates that ultrasound-stimulated microbubble treatment has a supra-additive effect with radiation treatment *in vivo* in a large tumor model. Human PC3 tumor xenografts in chemically immunocompromised rabbits were treated with single- and multi-fraction USMB and radiation treatments over a period of 3 weeks with an enhancement of the radiation response. Histological and immunohistochemical analysis indicated a statistically significant vascular disruption that was detected 24 hours after single-dose combined (USMB and radiation) treatments. This was linked to cell death and reductions to blood flow from *in vivo* imaging, but not to significant changes in oxygenation, suggesting that a single treatment may avoid creating hypoxic regions in the tumor microenvironment. Results here further indicate that a fractionated treatment schedule involving five fractions/week of 2 Gy, combined with two fractions/week of USMB treatments can inhibit tumor growth and even promote tumor regression. Combined fractionated treatments had a statistically significant interactions in the reduction of tumor growth rate and total tumor size during the observed period.

This study agrees with previous data suggesting the use of a single USMB treatment as a mechanoacoustic method of enhancing radiation response. Immunohistochemistry assessments after 24 hours from therapy in the larger tumor model used here, shown in Fig 1, display tumor cell death in H&E and ISEL-staining, which was most prevalent in a single 8 Gy and combined 8 Gy and 1% (v/v) USMB treatments. The percentage of cell death in ISEL staining was quantified (Fig 1C), which indicated that treatment groups had a significant interaction with respect to cell death, and combined treatments had significantly higher cell death compared to the control and USMB-only treatment groups. Additionally, there was a significant increase in vascular disruption assessed through staining for Factor VIII leakage in single USMB and 8 Gy treatments, as well in the combined treatments after 24 hours (Fig 2A). The quantified vascular counts indicated that all group-wise comparisons were significantly different apart from USMB-only and radiation-only treatments, suggesting that the combined approach using USMB prior to radiation is required to potentiate the increased vascular damage observed (Fig 2B).

Previously reported cell death levels from ISEL staining from combined single fraction treatments of USMB and 8 Gy radiation on human PC3 xenografts in mice resulted in enhancements of 49 to 70% when combined [21, 31, 41]. The fraction of cell death for the combined single fraction treatments exist up to 50% in this study, which were lower than previous results in mice. The extent of cell death, confirmed by the white blanched areas on H&E images (Fig 1A) and its associated ISEL positive staining, is mainly limited to the periphery region. Vascular disruption was assessed over the whole tumor at a higher magnification with high specificity (Fig 2C), suggesting more heterogeneous tumors may benefit from spatially focused USMB to extend the cell death to the central region. To maximize tumor cell kill, we utilized results from previous single-exposures of USMB and 8 Gy radiation in murine models. For example, an 8-fold increase in tumor cell kill and 14-fold increase in ceramide production was found using a peak negative pressure of 570 kPa and 1% (v/v) concentrations of microbubbles [35].

The temporal delivery of treatment was investigated by partitioning animals into single fraction cohort, and a multi-fraction cohort. The single fraction experiments used a larger radiation dose of 8 Gy and was performed immediately after USMB delivery, as this is within the time window (≤ 6 h) for maximal synergistic effects [21]. Treatments involving multiple fractions of combined therapy had more apparent arrest of tumor growth and survival using rabbit death and human-modified endpoints and was the only treatment group to demonstrate a trend in where there was arrested tumor growth after 3 weeks (Fig 4). The surviving fraction from combined treatments was similar in comparison to previous observations in PC-3 and MDA-MB-231 tumor bearing mice after 21 days that received lower fractionated doses [21, 42], though our results indicate the twice-weekly USMB exposure alone had less effect. The difference in tumor size compared to the mouse model likely plays a role in the decreased induction of endothelial cell damage caused by the USMB treatment, as the acoustic field was applied uniformly towards the more superficial area of the tumor. Analysis (log-rank) revealed significant differences between the surviving fractions for each group by the third week of experimentation (Fig 5), however, treatment outcome in larger tumor models and human tumors may benefit from increasing the endothelial damage caused by the mechanical stimulation of microbubbles. This study used twice-weekly USMB therapies, either alone or in combination with fractionated 2 Gy doses. The USMB schedule corresponds to the 1st and 5th fractions each week. Maximal tumor growth delay from single USMB exposures were previously shown to be 5 days [21]. The treatment schedule here ensures repeated USMB treatments do not exceed this time frame. Future work involving fractionated USMB therapy could increase the frequency of treatments to the same time points as conventional fractionated XRT (from twice to five times weekly), or target deeper vasculature using Focused Ultrasound (FUS).

In this study, the fractionated cohort used a similar treatment schedule to that of conventionally fractionated RT using daily doses of 2.0 Gy. These doses are associated with increased perfusion and reduced hypoxia, as well as maturation of endothelial vessels through pericyte recruitment [13, 43]. In contrast, higher doses per fraction from (i.e, 15 fractions of 4 Gy) have been shown to cause endothelial cell death and reduced microvascular density [44]. This is demonstrated here as intra-tumor endothelial damage was significantly higher after single doses of combined 8 Gy radiation and USMB after 24 h. In the conventionally fractionated 2 Gy doses with combined USMB, there were no significant differences in tumor sizes by the end of the first week with similar biologically equivalent radiation doses. However, by the end of the 2nd week (10th fraction and four USMB treatments), the combined USMB+XRT treatment group displayed significant differences compared to untreated controls or USMB-alone. Anti-tumor effect and is also observed by using similar staining techniques to the single-dose cohort, as combined treatments have illustrated enhanced tumor cell death associated with vascular disruption (Fig 6). Though, there were fewer relative differences observed in endothelial cell staining for combined fractionated treatment delivery (Fig 6, right column) compared to combined single-dose treatments (Fig 2). The observation period may account for these differences as vessel normalization can occur after fractionated therapy, or there is less ceramide production after each USMB + 2 Gy fraction. Additionally, an increase in fibrosis over the long term was detected in the tumors receiving the combined treatment. Fibrosis is a repair process that occurs after cell death, in which dying cells are replaced by excess fibrous connective tissue. Here, fibrosis increased with the fractionated treatments (Fig 7). The increase in fibrosis in the fractionated combined treatments compared to XRT-only more closely resemble single-dose therapies, as shown with 10–30 Gy single doses resulting in increased collagen content 5 days after therapy [8]. Finally, as ISEL staining is effective prior to replacement fibrosis, the results here from trichrome suggest similar cell kill and tumor control is achieved. The relative changes by treatment group for tumor cell kill from higher, single-dose treatments (Fig 1C) strongly correlate to the conventionally fractionated treatments by the end of the third week (Fig 7C).

In contrast to previous studies, several notable differences were observed. 3D Doppler detected blood flow at 24 hours was reduced for all single-dose treatment groups as expected (Fig 3A & 3C). Previous studies in mice have shown similar effect, with reported decreases in the vascular index 45% to 89% at combined 8 Gy and USMB therapies [36, 40]. This study used a centre frequency of 21 MHz compared to 25 MHz by Kwok *et al*. who were the only ones with similar decreases in VI. The reduction in frequency decreases the sensitivity towards smaller vessels < 0.75 mm/s [45], which may bias our measurements towards changes in larger vasculature in the tumor. For example, Fig 3A demonstrates that vascular reduction is mainly limited to the peripheral regions with diffuse areas remaining in the centre. Combined USMB and radiation has been explored in other larger tumor models, for example in a rat model for hepatocellular carcinoma. A 70% decrease in intra-tumor vascularity was reported, as measured through power Doppler imaging [39]. This intermediate decrease in vascular disruption suggests that tumor volume and heterogeneity may play a role in outcome. For larger tumor models then, the trade-off between flow sensitivity and tumor coverage may need to be optimized further to display differences between treatment groups for *in vivo* monitoring of treatment efficacy. Finally, this study also demonstrated no significant decreases in intra-tumor oxygenation, measured through photoacoustic imaging (Fig 3D). Stability in intra-tumor oxygenation has also been reported in larger tumor models after combined USMB+XRT therapy. The decreases in tumor oxygenation that have been observed in mice may have been driven by anoxia from high radiation-induced cell kill at these tumor volumes.

In summary, this work validates the efficacy of using the acoustic stimulation of microbubbles to sensitize tumor cells to radiation therapy in a rabbit model. Results indicate that USMB combined with radiation therapy results in tumor vascular disruption, leading to tumor cell death. This treatment modality is particularly advantageous because it allows the safe targeting of tumor cells and vasculature. Furthermore, it enhances the effect of radiation therapy, allowing the use of lower doses in order to minimize the negative effects to surrounding healthy tissues.
